# Emergent Endoscopy for Esophageal Foreign Body Removal: The Impact of Location

**DOI:** 10.7759/cureus.21929

**Published:** 2022-02-05

**Authors:** Babak T Sagvand, Daniel Najafali, Isha Yardi, Iana Sahadzic, Leenah Afridi, Alyssa Kohler, Ikram Afridi, Noorvir Kaur, Quincy K Tran

**Affiliations:** 1 Emergency Medicine and Critical Care, University of Maryland School of Medicine, Baltimore, USA; 2 Emergency Medicine and Critical Care, Carle Illinois College of Medicine, Urbana, USA

**Keywords:** location of endoscopy, food impaction, esophageal foreign body, endoscopy, emergent endoscopy

## Abstract

Background

Timely intervention is essential for the successful removal of ingested foreign bodies. Emergent endoscopy (EGD) is usually performed in the emergency department (ED), operating room (OR), intensive care unit (ICU), or endoscopy suite. However, because the endoscopy suite is not always available, this study investigated the impact of location outside of the endoscopy suite on the successful removal of ingested foreign bodies and other patient outcomes.

Methodology

We reviewed charts of patients who underwent EGD for foreign body removal at an academic quaternary center between January 01, 2012, and December 31, 2020. We defined successful EGD as retrieval of the foreign body at the first attempt and not requiring subsequent endoscopy or surgical intervention. We performed descriptive and inferential statistical analyses and conducted classification and regression trees to compare endoscopy procedure length (EPL) and hospital length of stay (HLOS) between different locations.

Results

We analyzed 77 patients, of whom 13 (17%) underwent endoscopy in the ICU, 46 (60%) in the OR, and 18 (23%) in the ED. Endoscopic removal failed in four (5%) patients. Endoscopy length was significantly shorter in the OR (67 (48-122) minutes) versus the ICU (158 (95-166) minutes, *P* = 0.004) and the ED (111 (92-155) minutes, *P *= 0.009). Time to procedure was similar if the procedure was performed in the ED (278 minutes), the ICU (331 minutes), or the OR (378 minutes). The median (interquartile range) of HLOS for the OR group (0.87 (0.54-2.03) days) was significantly shorter than the ICU group (2.26 (1.47-6.91) days, *P* = 0.007).

Conclusions

While performing endoscopy for esophageal foreign body removal in the OR may be associated with a shorter EPL and HLOS, no location was inferior for overall outcomes. Further prospective and randomized studies are needed to confirm our findings.

## Introduction

Patients who accidentally ingest foreign bodies typically present to an emergency department (ED) for care. The management of ingested foreign bodies involves a multidisciplinary approach, including emergency nurses, emergency clinicians, gastroenterologists, and otolaryngologists [1]. Although foreign body ingestion is most common among children, it also occurs in adults with pre-existing esophageal pathology, patients with a psychiatric history, or individuals who are impaired by alcohol intoxication [2,3]. Ingestion of sharp foreign bodies, batteries retained in the esophagus, and complete esophageal obstruction due to food impaction resulting in the inability to handle secretions are major indications for emergent endoscopy (EGD), which is defined as endoscopy within two hours of arrival at an ED [4]. Failure in the timely removal of sharp foreign body ingestion or food impaction can result in serious complications, such as esophageal perforation, obstruction, bacteremia, aortoesophageal fistula, and tracheoesophageal fistula formation [5-9].

A 2012 narrative review of foreign body ingestion in adults suggested that approximately 80% of all foreign bodies pass naturally without any gastrointestinal intervention [10]. For the remaining patients who require intervention, factors that affect the successful removal of ingested foreign bodies include the type, shape, anatomic location of the ingested foreign body; the time interval from ingestion to presentation; the number of foreign bodies present; equipment quality; and any concurring complications, such as perforation, bleeding, or infection before endoscopy [11]. Gastrointestinal endoscopic procedures are primarily performed in an endoscopy suite with dedicated anesthesiologists, nurses, and technicians [12]. However, when timely endoscopy in the endoscopy suite is not feasible, these procedures are commonly performed in secondary locations, such as the intensive care unit (ICU), the ED, and the operating room (OR). In all three locations, patients can receive sedation, cardiopulmonary monitoring, and, if necessary, endotracheal intubation and mechanical ventilation.

The secondary location selected for emergent endoscopies may affect the length of the procedure in an already time-sensitive protocol; however, data regarding the effect of secondary locations on endoscopy outcomes are scarce [13]. Current literature provides no universal consensus on how to choose between these secondary locations when necessary. Published practice guidelines on the removal of foreign bodies, such as the official statement released by the European Society of Gastrointestinal Endoscopy, mention recommendations on the time-sensitive nature of foreign body removal and other factors such as imaging protocols but have no specific evidence-based or outcomes-based recommendations on the location of where an endoscopy should be performed [4]. The landscape of literature on endoscopies for esophageal foreign body removal focuses primarily on examining the efficacy of various endoscopy protocols, such as comparing rigid versus flexible endoscopy techniques, or identifying predictive parameters for endoscopies following foreign body ingestion [9,14]. Most observational studies on esophageal foreign body management focus on endoscopy cases performed in traditional endoscopy suites or the OR. There has been very little data on emergent endoscopies performed in EDs and ICUs. Additionally, during the coronavirus disease 2019 (COVID-19) pandemic when hospitals were understaffed, emergent endoscopies were performed in various locations and were dependent on anesthetic support without a clear understanding of the impact of performing endoscopies in secondary locations [15].

Our study aimed to investigate the impact of location on endoscopic outcomes, such as successful removal rate and timing of intervention. We also investigated other patient-centered outcomes such as the endoscopy procedure length (EPL) and patients’ hospital length of stay (HLOS).

## Materials and methods

Patient selection and study design

This was a single-center, retrospective review of consecutive adult patients who underwent emergent upper endoscopy for foreign body ingestion at our academic quaternary medical center over a nine-year period between January 2012 and December 2020. All adult patients undergoing upper endoscopy for esophageal foreign bodies were reviewed. Patients were included in our analysis if they underwent an EGD in the ED, OR, or ICU for retained sharp foreign body in the esophagus or esophageal food impaction resulting in the patient not being able to manage their secretions. Other patients with foreign body ingestion or food impaction who did not meet the above criteria (such as blunt object, object in stomach, etc.) were excluded. Additionally, we excluded patients with a rectal foreign body, those undergoing percutaneous endoscopic gastrostomy tube placement, pregnant women, or those with missing records. The study was approved by our institutional review board (IRB, study number HP-00084554).

Study setting

Our institution is a quaternary medical center with gastroenterology coverage 24 hours a day, seven days a week. The gastroenterology service manages patients who present to our medical center or patients who are transferred from other hospitals within the region. Our endoscopy suite is fairly busy, and the schedule for the endoscopy suite is frequently set well ahead of time. As a result, EGD procedures, as determined by our gastroenterologists, are mostly performed in the ED, OR, or ICU to avoid delays in timely interventions. The clinical policy at our institution is that we observe patients who undergo an EGD for a sharp foreign body in the esophagus or food impaction causing inability to manage secretions post-procedure. This is mostly for timely diagnosis of possible complications, such as microesophageal perforation and the ability to tolerate oral intake.

Data collection and management

Data were extracted from patients’ charts and recorded into a secure standardized Microsoft Excel Database (Microsoft Corp, Seattle, WA, USA) by research team members who were blinded to the study hypothesis. Before data collection, research team members were trained by the senior investigator for data collection. Training included sets of five patients until 90% of the research team members’ data agreed with the senior investigator’s data. Any disagreements in data collection were adjudicated through discussion among the junior investigators and the principal investigator. Study data were collected from multiple sources, including procedure notes, ED charts, ICU flowsheets, and our institution’s electronic health records. We collected data on each patient’s medical history, type of foreign body and location, intentional ingestion, radiographic visibility, and time of endoscopy. Other data that was obtained included time to procedure, procedure length (EPL), HLOS, whether the patient was transferred from another hospital, any medications administered, and any complications. We defined any complications as perforation of any viscus, infection, etc.

Outcome measures

The primary outcome of interest was the success rate of foreign body removal stratified by the location of the endoscopy procedure (e.g., ED vs. OR vs. ICU). An endoscopy was determined successful if the foreign body was removed after the first attempt and required no further endoscopy or surgical intervention. Secondary outcomes of interest were compared for endoscopies in each of the three locations and included: (1) time interval from presenting at our medical center to start of endoscopy, (2) length of endoscopy, defined as the duration from the start of the procedure to the time the scope was withdrawn from patients, as specified in the procedure notes, and (3) HLOS, defined as the time interval between admission to discharge of patients from our academic medical center.

Statistical analysis

Descriptive analysis was performed to describe the characteristics of patients undergoing emergent upper endoscopy. We reported continuous patient characteristics as mean (±standard deviation, SD) or median (interquartile range, IQR). We assessed parametric continuous data using Student’s t-tests and nonparametric continuous data using Mann-Whitney U tests. Categorical data were reported as frequencies and percentages, N (%), and compared using either Pearson’s chi-square test or Fisher’s exact test when appropriate.

Before performing our analysis, we assessed the histograms of the continuous outcomes of interests (e.g., EPL and HLOS). We subsequently dichotomized these continuous dependent variables according to their distribution frequencies. Classification and regression trees (CART) analysis was conducted to demonstrate variables associated with emergent upper endoscopy and to develop a predictive model for the outcomes of interest. This nonparametric technique uses decision trees in a flowchart-like manner to classify the cohort based on binary decision points and has been shown to be particularly valuable for datasets with missing variables and outliers [16,17]. For CART analysis, a 10-fold cross-validation method was used to assess the reliability of variables as predictors. Through recursive partitioning, the model places the study population into a series of dichotomous splits (e.g., HLOS ≥one day or not, EPL ≥two hours or not), and then evaluates each independent variable (e.g., demographics and clinical characteristics) to maximize its sensitivity and specificity for the precise classification. Terminal nodes signify the final branch points significant to the outcome of interest. Variable importance measures model improvement when splits are made on a predictor. Relative variable importance of 100% is assigned to the predictor most strongly associated with the outcome of interest. These relative variable importance values determine the predictive values of an independent variable, but not the order of appearance in the decision tree. Once the analysis is complete and the best split for every variable is determined, the algorithm easily communicates the findings by dichotomizing patients into two separate homogenous groups. The CART analysis has certain advantages over logistic regressions because the CART analysis allows for an easy interpretation of the decision tree and its relative variable importance. Furthermore, the CART analysis provides a better description of the association of continuous independent variables because it provides clear cut-off values, which are particularly valuable clinically. We assessed the goodness-of-fit of the CART model via area under the receiver operating characteristic curve (AUROC) analysis. The goodness-of-fit for the model improves as the AUROC approaches one.

All statistical tests were performed using Minitab version 19 (www.minitab.com; Minitab LLC, State College, PA, USA). Independent variables with a two-tailed P-value of <0.05 were considered statistically significant.

## Results

Demographics and clinical characteristics of emergent upper endoscopy patients

**Figure 1:**
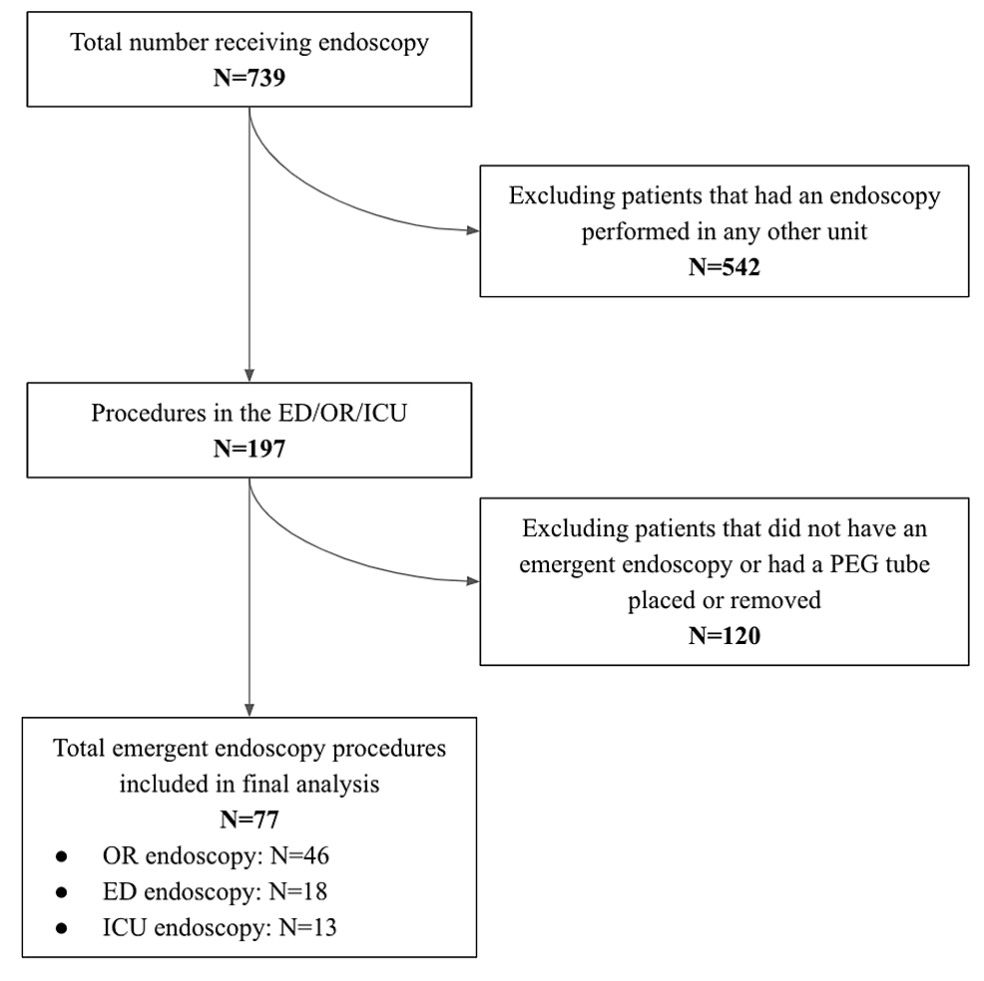
Patient selection diagram mapping EGD patients included in the final analysis. ED: emergency department; EGD: emergent endoscopy; ICU: intensive care unit; OR: operating room; PEG: percutaneous endoscopic gastrostomy

In total, 739 patients underwent upper endoscopy at our academic medical center during the study period. There were 542 patients who underwent upper endoscopy in clinical settings other than the ED, OR, or ICU. Among the remaining 197 patients, 120 had scheduled, non-emergent endoscopies. These patients were excluded from the study. A total of 77 patients were identified as having EGD in the ED, OR, or ICU and were included in the final analysis (Figure 1).

We stratified patients based on the location of their procedure. Of our cohort, 13 (17%) emergent endoscopies were performed in the ICU, 46 (60%) in the OR, and 18 (23%) in the ED. The mean age (SD) was 37 (25) years, the median (IQR) body mass index (BMI) was 26 (20-31) kg/m^2^, and 28 (36%) patients were female. Before the procedure, 45 (58%) patients had radiographic visibility of the ingested foreign body on imaging studies, and the majority of the procedures (65%) were performed on a weekday. Underlying comorbidities were seen in 45 (58%) patients, the most predominant of which were known psychiatric history (32%) and esophageal pathologies (26%). Patients’ clinical information and characteristics, such as the specific foreign body and location, are summarized in Table 1.

**Table 1:** Demographic and clinical information of patients undergoing EGD. Bold cells indicate statistically significant variables (*P* < 0.05). BMI: body mass index; ED: emergency department; EGD: emergent endoscopy; ICU: intensive care unit; IQR: interquartile range; NA: not applicable; OR: operating room; PMHx: past medical history; SD: standard deviation

Variables	All patients	Location			P-value	P-value	P-value
		ED (A)	OR (B)	ICU (C)	(A vs. B)	(A vs. C)	(B vs. C)
Total patients, N	77	18	46	13	NA	NA	NA
Gender, N (%)							
Male	49 (64)	11 (61)	30 (65)	8 (62)	0.76	0.98	0.99
Female	28 (36)	7 (39)	16 (35)	5 (38)			
Age (years), mean (SD)	37 (25)	46 (17)	30 (27)	51 (13)	0.006	0.42	<0.001
BMI (kg/m^2^), median (IQR)	26 (20–31)	30 (26–36)	23 (16–28)	30 (27–32)	0.004	0.62	0.022
PMHx, N (%)							
Esophageal pathologies	20 (26)	9 (50)	10 (22)	1 (8)	0.026	0.02	0.43
Psychiatric comorbidities	25 (32)	8 (44)	8 (17)	9 (69)	0.05	0.17	<0.001
Type of foreign body, N (%)							
Food bolus	39 (51)	10 (56)	23 (50)	6 (46)	0.69	0.61	0.81
Harmful object	11 (14)	5 (28)	2 (4)	4 (31)	0.016	0.99	0.018
Other	27 (35)	3 (17)	21 (46)	3 (23)	0.031	0.68	0.14
Foreign body location, N (%)							
Proximal esophagus	21 (27)	4 (22)	14 (30)	3 (23)	0.51	0.99	0.74
Mid-esophagus	9 (12)	1 (6)	8 (17)	0 (0)	0.43	NA	NA
Distal esophagus	14 (18)	7 (39)	4 (9)	3 (23)	0.008	0.45	0.17
Stomach	30 (39)	5 (28)	18 (39)	7 (54)	0.40	0.14	0.34
Duodenum	3 (4)	1 (6)	2 (4)	0 (0)	0.99	NA	NA
Radiographic visibility, N (%)	45 (58)	8 (44)	27 (59)	10 (77)	0.30	0.07	0.33
Intentional ingestion, N (%)	20 (26)	6 (33)	7 (15)	7 (54)	0.16	0.29	0.008
Time of endoscopy, N (%)							
Day (7:00–18:59)	47 (61)	6 (33)	35 (76)	6 (46)	0.001	0.47	0.08
Night (19:00–06:59)	30 (39)	12 (67)	11 (24)	7 (54)			
Weekday (Monday–Friday)	50 (65)	9 (50)	34 (74)	7 (54)	0.07	0.83	0.19
Weekend	27 (35)	9 (50)	12 (26)	6 (46)			
Weekday night	22 (36)	7 (39)	11 (24)	4 (31)	0.23	0.72	0.72
Weekend night	8 (38)	5 (28)	0 (0)	3 (23)	NA	NA	NA

Primary outcome: rate of successful foreign body removal

The primary outcome of interest was the overall success rate of foreign body removal stratified by the location of the endoscopy procedure. Foreign bodies were successfully removed on the first attempt in all but four (5%) patients. Of the four patients who required a repeat endoscopy, two (3%) endoscopies were performed successfully after one additional attempt, and two (3%) endoscopies were performed successfully after two additional attempts. All four endoscopies that required more than one attempt were completed in the ICU. Emergent endoscopies performed in the ED (*P* = 0.023) or OR (*P* = 0.002) were significantly more likely to be successful on the first attempt compared to endoscopies performed in the ICU. These results are presented in Table 2. However, there were not enough failed procedures to perform logistic regressions to identify independent variables that were associated with the successful removal of foreign bodies among different locations.

**Table 2:** Clinical features of patients undergoing EGD. Bold cells indicate statistically significant variables (*P* < 0.05). ED: emergency department; EGD: emergent endoscopy; HLOS: hospital length of stay; ICU: intensive care unit; IQR: interquartile range; NA: not applicable; OR: operating room

Variables	All patients	Location			P-value	P-value	P-value
		ED (A)	OR (B)	ICU (C)	(A vs. B)	(A vs. C)	(B vs. C)
Total patients, N	77	18	46	13	NA	NA	NA
Time to endoscopy (minutes), median (IQR)	334 (158–561)	278 (187–432)	378 (170–667)	331 (144–561)	0.28	0.87	0.65
Length of endoscopy (minutes), median (IQR)	89 (61–141)	111 (92–155)	67 (48–122)	158 (95–166)	0.009	0.44	0.004
HLOS (days), median (IQR)	1.24 (0.55–2.97)	1.37 (0.38–4.27)	0.87 (0.54–2.03)	2.26 (1.47–6.91)	0.55	0.09	0.007
Glucagon administration, N (%)	10 (13)	5 (28)	4 (9)	1 (8)	0.10	0.36	0.99
Hospital transfer, N (%)	25 (32)	4 (22)	15 (33)	6 (46)	0.41	0.25	0.51
Complications, N (%)	12 (16)	4 (22)	5 (11)	3 (23)	0.25	0.99	0.36
Previous attempts, N (%)							
No previous attempts	73 (95)	18 (100)	46 (100)	9 (69)	NA	0.023	0.002
One previous attempt	2 (3)	0 (0)	0 (0)	2 (15)	NA	NA	NA
Two previous attempts	2 (3)	0 (0)	0 (0)	2 (15)	NA	NA	NA

Secondary outcomes: time intervals from arrival to start of endoscopy, length of endoscopy, and hospital length of stay

Secondary outcomes of interest included time intervals from arrival to start of endoscopy, length of endoscopy, and HLOS. The median (IQR) time interval to start of endoscopy for all patients was 334 (158-561) minutes and was shorter in patients who underwent endoscopy in the ED (278 minutes), but this finding was not statistically significant across all three locations (ED, OR, and ICU; *P* > 0.05). The median procedural length of endoscopy for all patients was 89 (61-141) minutes. Across the three procedure locations, the median length of endoscopy was significantly shorter in the OR compared to the ED (67 (48-122) minutes vs. 111 (92-155) minutes; *P *= 0.009) and the ICU (158 (95-166) minutes; *P* = 0.004). However, there was no significant difference in length of endoscopy for procedures performed in the ED versus the ICU (*P* = 0.44).

The overall population’s median (IQR) HLOS was 1.24 (0.55-2.97) days, with patients who underwent the procedure in the OR (0.87 (0.54-2.03) days) having a significantly shorter HLOS compared to those who underwent endoscopy in the ICU (2.26 (1.47-6.91) days; *P *= 0.007). There was no statistically significant difference in HLOS when comparing the ED to the OR (*P* = 0.55) or comparing ED to the ICU (*P *= 0.09). Complication rates, hospital transfers, and glucagon administration were also similar across the three procedure locations (Table 2).

Predictors of endoscopy procedure length

**Figure 2:**
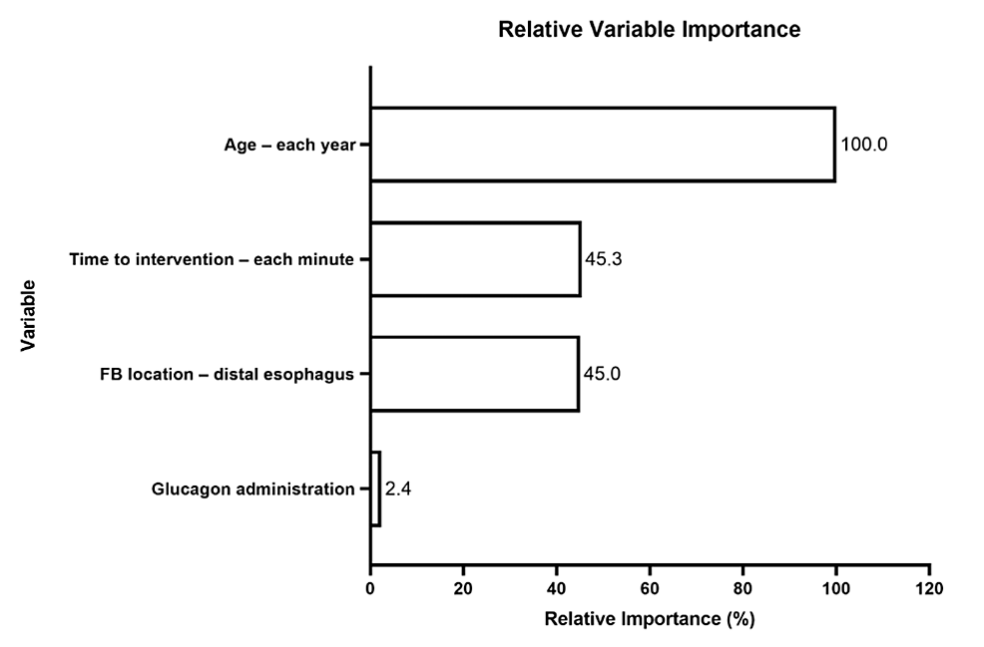
Relative variable importance for the outcome of EPL. Note: Variable importance measures model improvement when splits are made on a predictor. Relative importance is defined as the percentage improvement with respect to the top predictor. EPL: endoscopy procedure length; FB: foreign body

**Figure 3:**
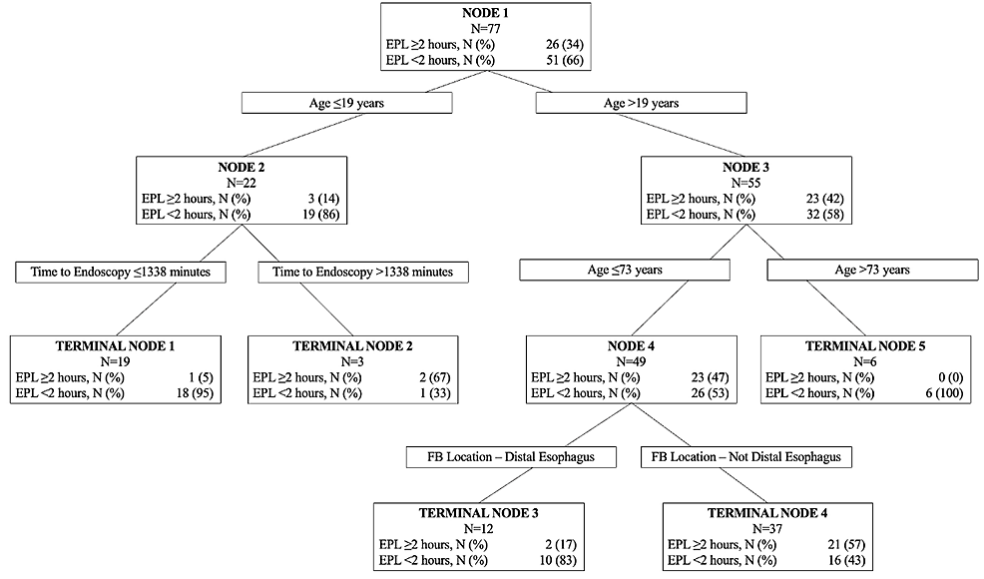
CART decision tree for the outcome of EPL. CART: classification and regression trees; EPL: endoscopy procedure length; FB: foreign body

Based on the histogram, EPL was dichotomized as being a short procedure (<two hours) or a long procedure (≥two hours). In the CART model, we identified age as the most important variable for predicting EPL, followed by time intervals from arrival to start of endoscopy, and foreign body location (distal esophagus) (Figure 2). Age was the initial variable that split the tree with a threshold value of 19 years. For patients 19 years of age or younger, the next variable with significant interaction was the time interval from arrival to the start of procedure with a threshold of 1,338 minutes. This branch point resulted in two terminal nodes, those patients with lower (≤1,338 minutes) time to procedure (95% of patients) had a total procedure time of <two hours (Figure 3, Terminal Node 1), whereas 67% patients with time to procedure of >1,338 minutes had a total procedure time of at least two hours or more (Figure 3, Terminal Node 2). Patients >19 years of age were further split into age older than 73, which resulted in a terminal node where all patients had a procedure length of <two hours (Figure 3, Terminal Node 5). For those patients younger than 73, foreign body location determined the next split resulting in two terminal nodes. Having a foreign body in the distal esophagus resulted in 83% of patients with a total procedure time of <two hours (Figure 3, Terminal Node 3), but having a foreign body located elsewhere led to 43% of patients having a total procedure time of <two hours (Figure 3, Terminal Node 4). The AUROC for this CART model was >0.7, with a sensitivity and specificity of >70%, which indicated that the independent variables fit the model well.

Predictors of hospital length of stay

**Figure 4:**
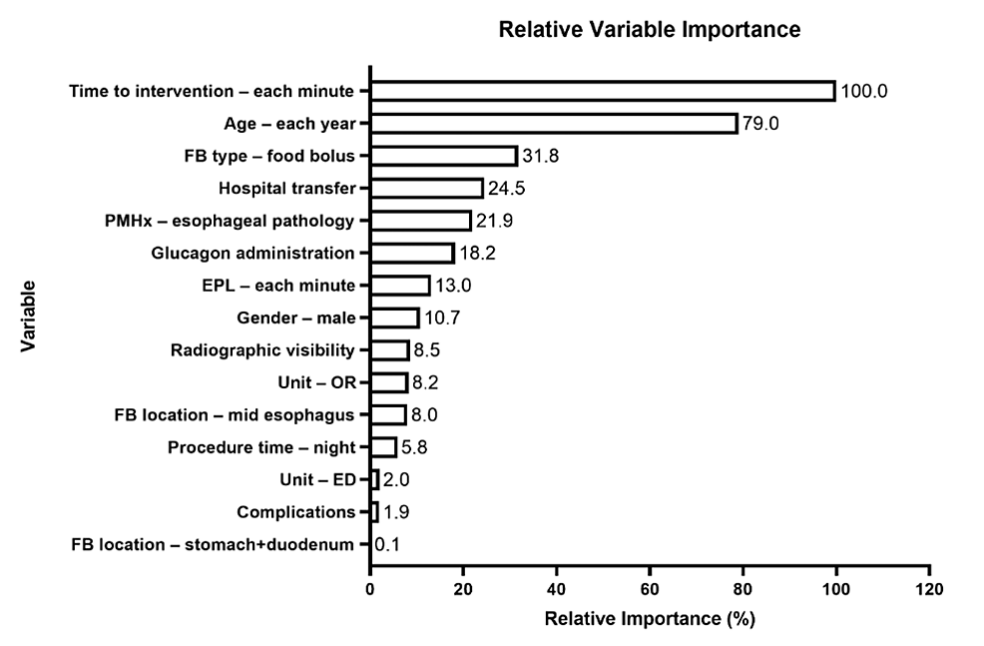
Relative variable importance for the outcome of HLOS. Variable importance measures model improvement when splits are made on a predictor. Relative importance is defined as the percentage improvement with respect to the top predictor. ED: emergency department; EPL: endoscopy procedure length; HLOS: hospital length of stay; FB: foreign body; OR: operating room; PMHx: past medical history

**Figure 5:**
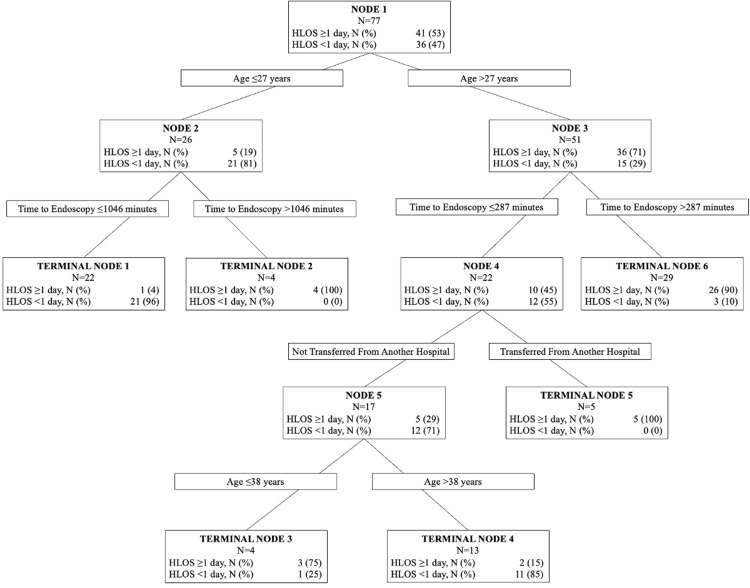
CART decision tree for the outcome of HLOS. CART: classification and regression trees; HLOS: hospital length of stay

We dichotomized HLOS as short stay (<one day) or long stay (≥one day) based on distribution frequencies. The CART analysis identified the time intervals to start of endoscopy (in minutes) as the most important factor for predicting HLOS, followed by age in years, foreign body type (food bolus), and hospital transfer status (Figure 4). In the CART model, age was identified as the variable in the primary split with a threshold value of 27 years (Figure 5, Node 1). Nineteen percent of patients 27 years of age or younger had an HLOS greater than one day. The time interval to start of endoscopy was identified as a significant interaction after both initial splits, with 1,046 minutes being the threshold value resulting in two terminal nodes for patients ≤27 years of age. Among those with a low (≤1,046 minutes) time interval to start of procedure, 4% of patients had an HLOS greater than one (Figure 5, Terminal Node 1), while none with a time to procedure >1,046 minutes had a short HLOS (Figure 5, Terminal Node 2). The threshold value for the next split after age was 287 minutes for patients >27 years of age resulting in a terminal node, with 90% of patients having HLOS greater than one day (Figure 5, Terminal Node 6). Among patients with a low (≤287 minutes) time interval to start of procedure, all patients transferred from another hospital had a longer HLOS (Figure 5, Terminal Node 5). For patients not transferred from another hospital, a threshold of 38 years of age resulted in two terminal nodes, with 75% (Figure 5, Terminal Node 3) and 15% (Figure 5, Terminal Node 4) of patients having HLOS greater than one day. The AUROC values for our model was >0.8, with a sensitivity and specificity of >80%, which indicated that the independent variables fit the model well.

## Discussion

Determining appropriate management for patients with esophageal foreign bodies is a common clinical dilemma. Our study was able to demonstrate that performing upper endoscopy in the ED or OR may be associated with a higher successful rate of foreign body removal. We further identified a few clinical predictors that were associated with other patient-centered outcomes such as EPL and patients’ HLOS.

Our study’s primary outcome was defined as successful endoscopic removal of foreign bodies. Endoscopic foreign body removal was successfully achieved at the first attempt in all but four patients. In all patients, the foreign body was eventually removed by repeat endoscopy and none required surgery. Hence, no further analysis was performed on the primary outcome due to the very low rate of failure. Instead, the secondary outcomes, including time to endoscopy, length of endoscopy, and length of hospital stay, were compared between groups.

Many previous studies have focussed on patients’ outcomes with respect to the timing of endoscopic intervention from the time of foreign body ingestion, devices used to extract the foreign body, anatomical location of the foreign body, type of foreign body, and characteristics of ingested foreign bodies [1,18-21]. However, in the case of EGD, it remains unclear whether specific locations where endoscopy is performed would affect patients’ outcomes. Recently, studies with EGD have found which predictors would affect emergent management and successful removal rate [14,22]. Moreover, focus has been given to endoscopy service practice patterns during the COVID-19 pandemic due to disruptions and staffing concerns [23]. Common indications for EGD include a sharp foreign body in the esophagus, batteries, and esophageal obstruction secondary to food. Coordinating an EGD can be challenging and resource-intensive [22]. It is a common practice to preemptively intubate patients with these conditions before endoscopy for airway protection during food or foreign body removal. Essentially, patients need cardiopulmonary monitoring, sedation, and intubation before scope insertion. These are all provided in an endoscopy suite, and it has been established that an experienced endoscopist can make a difference in outcomes [22]. However, endoscopy suites are usually operated on a previously set schedule. Additionally, in most centers, endoscopy suites are closed at night and on weekends. Hence, emergent endoscopic procedures are commonly performed in secondary locations, including the ED, OR, and ICU. Performing EGD at the bedside in the ED may seem appealing because it eliminates the need for transferring the patient to another location and can hypothetically reduce the time interval from arrival to the start of endoscopy. Scheduling a session in the OR is an additional challenging step, especially in busy centers. Transferring patients to the ICU can be favorable for procedures but is limited by bed availability. Tertiary academic centers tend to have higher ICU occupancy and less ICU availability [24]. Although transferring patients to the ICU adds transfer time with potential delays of intervention, once the patient is in the ICU, the procedure can be performed at the bedside and the same ICU team provides pre and postprocedure care. In this study, we demonstrated that performing endoscopy in the ICU is associated with fewer successful attempts in our inferential analyses; however, our CART analysis did not identify location as a predictor for other patient-centered outcomes. Further studies are needed to confirm our observation.

There was no statistically significant difference in the time intervals from arrival to start of endoscopy between the secondary locations. Although performing endoscopy at the bedside in the ED eliminates the need for transferring patients to another location, this did not translate to a significantly shorter time to endoscopy. Performing a bedside procedure in the ED requires an emergency medicine physician to allocate time to initiate sedation and intubate the patient. This can be a potential source of delay if there are multiple patients requiring emergent attention or when the ED is busy. A patient’s nurse is also required to stay in the room for monitoring and drug administration throughout an occasionally lengthy procedure, which can further interfere with the workflow of a busy ED. These requirements will place significant strains on overcrowded EDs. Therefore, we suggest that EGD should be performed in the ED as a last resort.

Patients who underwent endoscopic removal of a foreign body in the ICU were associated with increased HLOS compared to those undergoing the procedure in the OR. The reason was likely because patients are usually transferred to a regular medical ward from the ICU prior to discharge, which can potentially add to HLOS. This can also be affected by the availability of the ward. The majority of patients with a sharp foreign body in the esophagus or esophageal food impaction do not have ICU needs once the foreign body has been removed.

Endoscopic foreign body removal in the OR was associated with a shorter length of procedure compared to both the ED and ICU. The presence of a dedicated anesthesiology team can potentially result in a higher quality of sedation, a still patient, and, subsequently, a more efficient endoscopy. Furthermore, the OR hypothetically provides a more controlled environment with potentially fewer bystanders, minimizing distraction for the endoscopist. As a result, performing the procedure in the OR appeared to provide the best overall outcome when compared to the ED or the ICU.

Limitations

Our study has several limitations. This study was retrospective and included a relatively small cohort of patients due to the nature of the condition. Our final analysis had even smaller groups for each location. However, the sample size of our study (N = 77 vs. N = 67) was slightly larger than a previous study focusing on EGD for foreign body removal [22]. We did not have enough failure of removal to perform statistical analysis for this outcome, which may be indicative of the quality of endoscopic expertise at our institution. Due to the retrospective nature of our study, the specifics of each clinical scenario and logistical factors that resulted in deciding to perform endoscopic foreign body removal in the OR, ED, or ICU could not be identified. Because we only had a few cases in 2020, the impact of the COVID-19 pandemic and staff shortage on outcomes of patients who ingested foreign bodies remains unknown. Despite these limitations, our exploratory study provides insight into the influence that the location of endoscopy may affect patient outcomes. Furthermore, our study also provided predictors for patient-centered outcomes that previous studies did not investigate and further information for future investigations about this important topic.

## Conclusions

While performing endoscopy for esophageal foreign body removal in the OR may be associated with a shorter length of procedure, no location was inferior in terms of the overall outcomes. Endoscopic intervention in the ICU did not result in better outcomes than the ED or OR and was associated with increased HLOS, at least compared to those who received their procedure in the OR. Until further studies are available to confirm our observations, clinicians should consider the OR as the first choice for endoscopy location when resources are available.
